# Stress CT myocardial perfusion imaging after arterial switch operation for transposition of the great arteries

**DOI:** 10.1093/ehjimp/qyag139

**Published:** 2026-08-25

**Authors:** Christos Gkizas, Benjamin Longere, Olivia Domanski, Aimee Rodriguez-Musso, Mehdi Haidar, Mariia Tregubova, Cedric Delhaye, Cedric Croisille, Francois Godart, Francois Pontana

**Affiliations:** Department of Cardiovascular Imaging, Heart and Lung Institute, University Hospital of Lille, Bd of Prof Jules Leclercq, Lille 59000, France; Department of Cardiovascular Imaging, Heart and Lung Institute, University Hospital of Lille, Bd of Prof Jules Leclercq, Lille 59000, France; INSERM UMR 1011, Institute Pasteur of Lille, EGID (European Genomic Institute for Diabetes), FR3508, Univ Lille, Lille 59000, France; Paediatric and Congenital Cardiology Department, Heart and Lung Institute, University Hospital of Lille, Lille 59000, France; Department of Cardiovascular Imaging, Heart and Lung Institute, University Hospital of Lille, Bd of Prof Jules Leclercq, Lille 59000, France; Department of Cardiovascular Imaging, Heart and Lung Institute, University Hospital of Lille, Bd of Prof Jules Leclercq, Lille 59000, France; Department of Cardiovascular Imaging, Heart and Lung Institute, University Hospital of Lille, Bd of Prof Jules Leclercq, Lille 59000, France; Department of Cardiology, Heart and Lung Institute, University Hospital of Lille, Lille 59000, France; Department of CT Clinical Research, Siemens Healthineers, Bordeaux 33600, France; Paediatric and Congenital Cardiology Department, Heart and Lung Institute, University Hospital of Lille, Lille 59000, France; Department of Cardiovascular Imaging, Heart and Lung Institute, University Hospital of Lille, Bd of Prof Jules Leclercq, Lille 59000, France; INSERM UMR 1011, Institute Pasteur of Lille, EGID (European Genomic Institute for Diabetes), FR3508, Univ Lille, Lille 59000, France

**Keywords:** Arterial switch operation, Transposition of the great arteries, Computed tomography, Myocardial perfusion imaging, Coronary angle, neo-aorta

## Abstract

**Aims:**

Coronary abnormalities are late complications of arterial switch operation for transposition of the great arteries (ASO-TGA) and may remain silent for decades. We evaluated the feasibility and diagnostic performance of stress CT myocardial perfusion imaging (CT-MPI) in adults after ASO.

**Methods and results:**

Consecutive adults referred for clinically indicated coronary CT angiography (CCTA) underwent regadenoson stress CT-MPI using either dynamic energy-integrating detector CT (EID-CT) with quantitative myocardial blood-flow mapping or static photon-counting detector CT (PCCT) with qualitative and semiquantitative iodine–map assessment. All patients underwent stress cardiac magnetic resonance imaging (CMR) and invasive coronary angiography (ICA) within 3 months. Stress CMR served as the functional reference standard and ICA as the anatomical reference standard. Thirty-six patients were included (29 men; median age, 33.5 years), with 18 examined using each CT-MPI technique. Median neo-aortic diameter was 42 mm. At the per-segment level, CT-MPI achieved 95.5% sensitivity, 99.6% specificity, and 99.5% accuracy vs. stress CMR. In technique-stratified coronary–territory analyses, accuracy was 98.1% for static PCCT-MPI and 100% for dynamic EID-CT MPI. Against ICA, per-vessel sensitivity, specificity, and accuracy for obstructive coronary artery disease were 70.0%, 100%, and 98.6%, respectively. Coronary stenosis and a smaller coronary take-off angle were associated with ischaemia, whereas aortic dimension was not. A negative CT-MPI could have obviated additional testing in 30 of 36 patients (83.3%).

**Conclusion:**

Stress CT-MPI combined with CCTA was feasible and demonstrated high diagnostic performance for detecting myocardial ischaemia after arterial switch operation. Larger prospective studies are needed to confirm these findings and define its clinical role.

## Graphical Abstract

**Graphical Abstract:**
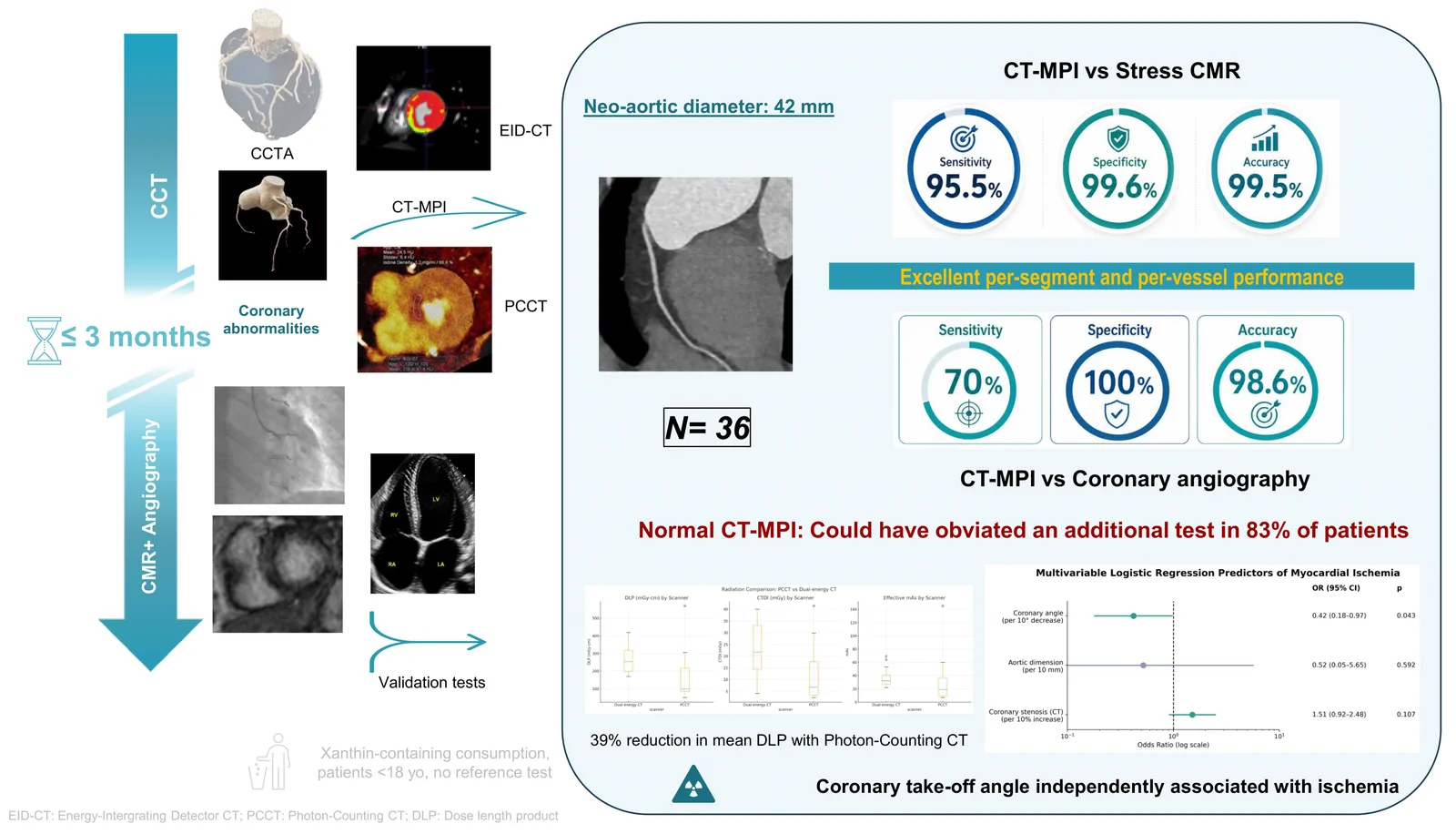

## Introduction

The arterial switch operation (ASO) for dextro-transposition of the great arteries (d-TGA) provides excellent survival into adulthood.^1,2^ However, lifelong imaging surveillance is performed periodically to detect late complications such as neo-aortic valve dysfunction, pulmonary artery stenosis, ventricular dysfunction, and coronary abnormalities.^3,4^ Echocardiography is the first-line examination, while cardiac magnetic resonance imaging (CMR) serves as an excellent baseline and follow-up method for functional and anatomical assessment.^3,4^

Less is known about the long-term patency of the translocated coronary arteries and the occurrence of myocardial ischaemia during adulthood. Potential mechanisms of myocardial ischaemia after coronary translocation include coronary kinking, anatomical distortion, stretching, or extrinsic compression.^5^ Unusual anatomical coronary patterns or progressive enlargement of the neo-aorta over time may alter proximal coronary geometry and predispose to higher risk of ischaemia.^5,6^ Due to cardiac denervation resulting from dissection of the cardiac plexus during surgery, ASO-TGA patients with coronary artery stenosis often remain asymptomatic for many years despite preserved ventricular function.^1–5^ However, long-term follow-up studies have reported significant coronary stenosis in asymptomatic children and adult patients with repaired d-TGA.^1–6^

Current imaging strategies primarily focus on anatomical assessment of the coronary arteries. Coronary CT angiography (CCTA) provides excellent spatial resolution and is widely used to evaluate coronary reimplantation and detect high-risk anatomical features such as intramural coronary course or acute coronary angulation. However, anatomical imaging alone does not necessarily establish the physiological significance of these findings.^7,8^ Modern guidelines recommend several functional tests for ischaemia evaluation after ASO, although these modalities demonstrate variable sensitivity and specificity.^6–11^ Moreover, there is no clear consensus regarding the optimal modality or frequency of testing in asymptomatic patients, even when anatomical or functional abnormalities are identified on echocardiography. In addition, the performance and interpretation of these tests often depend on institutional expertise and the availability of multidisciplinary congenital heart disease teams.

Stress CT myocardial perfusion imaging (CT-MPI) has recently emerged as a promising technique that allows simultaneous evaluation of coronary anatomy and myocardial perfusion within a single examination.^12,13^ This integrated approach may be particularly relevant in patients with repaired d-TGA, in whom coronary geometry and neo-aortic root enlargement have been proposed as potential contributors to ischaemia risk.^5–7,14^ However, the role of CT-based perfusion imaging in congenital heart disease populations has not yet been established.

We therefore evaluated the feasibility and diagnostic performance of stress CT-MPI combined with CCTA in adults after ASO, using stress CMR as the established functional reference standard for myocardial ischaemia and invasive coronary angiography (ICA) as the anatomical reference standard for obstructive coronary artery disease, its potential to reduce downstream diagnostic testing, and the relationship between ischaemia and anatomical markers such as coronary take-off angle and neo-aortic diameter.

## Methods

**Figure 1:**
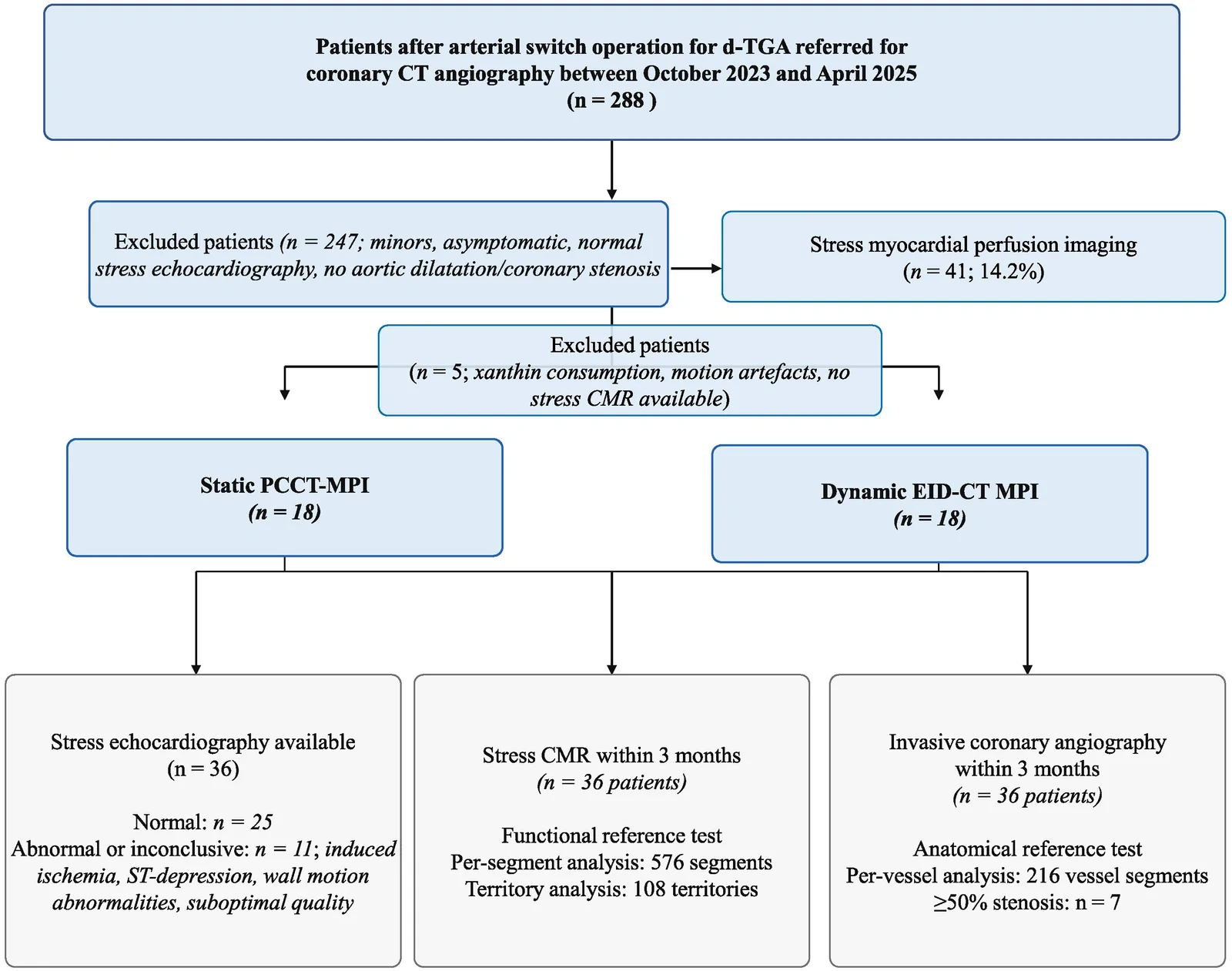
Flowchart of the study.

This single-centre, retrospective study was approved by the Institutional Review Board (CRM-2603-553), and written informed consent was obtained during the initial clinical care. Between October 2023 and April 2025, consecutive adult patients referred for clinically indicated CCTA underwent stress CT-MPI using either a first-generation dual-source photon-counting CT scanner (PCCT; NAEOTOM Alpha, Siemens Healthineers) or a third-generation dual-source energy-integrating detector CT scanner (EID-CT; SOMATOM Force, Siemens Healthineers). No patient underwent both CT-MPI techniques. Stress CMR and ICA were subsequently performed in all patients within 3 months of CT-MPI, with median intervals of 9 days (range: 1–64 days) and 28 days (range: 2–82 days), respectively. CT myocardial perfusion imaging readers were blinded to the findings of stress CMR and ICA. *Figure 1* shows the flowchart of the study.

Patients were classified as d-TGA with intact ventricular septum, d-TGA with one or more ventricular septal defects, or Taussig–Bing anomaly. Aortic abnormalities included neo-aortic root dilatation, coarctation, interruption, or hypoplasia, while atrioventricular valves were considered abnormal if malattached, straddling, or overriding. Coronary anatomy was categorized as usual, single right (RCA) or left anterior descending (LAD) coronary artery, circumflex from the RCA, inverted coronaries, RCA from the LAD, intramural variants, or other atypical patterns.

Exclusion criteria were absence of indication for stress CT-MPI, xanthin-containing substance consumption within 24 h prior to the examination, contraindication to regadenoson, age < 18 years, or refusal to participate. Patients without at least one control functional testing within 3 months before or after stress CT-MPI were also excluded. Control functional tests considered included stress echocardiography, stress CMR, and ICA.

Coronary CT angiography was acquired using prospective high-pitch spiral triggering in patients with regular heart rates <65 bpm and prospective sequential triggering in those ≥65 bpm. For one patient, retrospective spiral gating was required due to arrhythmia. A 40- or 50-mL bolus of iobitridol (350 mgI/mL) at the same injection rate as for CT-MPI was administered for all CCTAs.

CT myocardial perfusion imaging was then performed at least 10 min after the CCTA acquisition per institutional practice using 400 µg of regadenoson (Rapiscan, GE Healthcare).^15,16^ Aminophylline was not routinely administered but could be given in cases of poor regadenoson tolerance.

To assess a myocardial fibrotic scar, late iodine enhancement (LIE) acquisitions were systematically performed 5 min after reinjection of 50 mL of the same contrast agent.

### EID-CT MPI protocol

Following intravenous administration of 400 µg of regadenoson, iodinated contrast material (50 mL) was injected at 6 mL/s once the heart rate had increased by ≥20 beats/min or 1 min after regadenoson administration, followed by a 40-mL saline chaser. Dynamic stress CT-MPI was initiated 5 s after contrast injection using an ECG-triggered shuttle-mode acquisition centred 250 ms after the R wave (end-systole). Whole-heart coverage was obtained every one to three cardiac cycles over approximately 30 s, yielding 10–15 perfusion phases. Acquisition parameters were as follows: detector collimation, 48 × 1.2 mm; tube voltage, 80 kVp; tube current-time product, 300 mAs; gantry rotation time, 0.25 s; and reconstructed slice thickness/increment, 3/2 mm. Late iodine enhancement imaging was subsequently performed in dual-energy mode at 80/150 kVp and 165 mAs.

### PCCT-MPI protocol

Static stress PCCT-MPI was performed 1–2 min after intravenous administration of 400 µg of regadenoson. Images were acquired at 120 kVp in QuantumPlus spectral mode (Siemens Healthineers), with a gantry rotation time of 0.25 s, automated tube-current modulation (CARE keV IQ Level and CARE Dose4D), an image-quality level of 100, and a 280-ms ECG acquisition window. Prospective high-pitch spiral triggering was used in patients weighing <85 kg, whereas prospective sequential triggering was used in those weighing ≥85 kg. To target peak myocardial wash-in, imaging was initiated 12 s after the start of a 40-mL bolus of iodinated contrast material (350 mgI/mL, iobitridol, Xenetix; Guerbet) administered at 3.5 mL/s. This fixed delay had previously been established from institutional dynamic CT-MPI time–attenuation curves obtained in 184 patients (mean, 12 ± 0.8 s; range, 9.7–13.3 s). Late iodine enhancement imaging was subsequently performed 5 min after reinjection of 50 mL of the same contrast agent, using prospective high-pitch spiral triggering in patients weighing <85 kg and prospective sequential triggering in those weighing ≥85 kg, with automated tube-current modulation and an image-quality level of 200.

### Image reconstruction and analysis

Energy-integrating detector CT MPI datasets were reconstructed with a 3-mm slice thickness and 2-mm increment and processed using commercial software (syngo CT Myocardial Perfusion, Siemens Healthineers). Motion correction was systematically applied before generation of myocardial time–attenuation curves. The arterial input function was sampled in the descending aorta and used to model contrast–medium kinetics and derive quantitative myocardial blood-flow (MBF) maps. The resulting colour-coded MBF maps were subsequently processed using prototype software (Cardiac Functional Analysis Prototype, Siemens Healthineers), which automatically generated 17-segment polar maps depicting MBF distribution within the subendocardial layer of the left ventricular myocardium. CCTA datasets were reconstructed with a 0.6-mm slice thickness and 0.6-mm increment using a Bv44 kernel and a 512 × 512 matrix.

PCCT-MPI series were reconstructed with a 0.4-mm slice thickness and 0.4-mm increment using a Qr44 kernel, a 512 × 512 matrix, and quantum iterative reconstruction at strength level 4. Coronary CT angiography datasets were reconstructed using the same slice thickness, increment, matrix, and iterative-reconstruction strength, with a Bv56 kernel in QuantumPlus spectral mode. Depending on the ECG-gating mode, additional cardiac phases were reconstructed when required. Late iodine enhancement datasets were reconstructed using the same parameters as PCCT-MPI, except for the use of a Qr32 kernel.

Quantitative MBF mapping is not available as a vendor-provided clinical application on the PCCT platform. Consequently, quantitative MBF values were derived only from dynamic EID-CT MPI, whereas static PCCT-MPI was assessed qualitatively and semiquantitatively using iodine maps and regional iodine–concentration measurements.

Regions of interest of 100–150 mm^2^ were placed on visually pathological and remote myocardium segments of the EID-CT MPI scans. Myocardial perfusion defects were defined as circumscribed areas with a relative MBF value (pathological/distant healthy myocardium) of 0.7 located on a coronary territory (13).

Definitions of remote and hypoattenuating myocardium on PCCT-MPI were established by consensus between two cardiovascular imagers (X.C. and B.L., with 9 and 14 years of experience, respectively).When a perfusion defect was present, the remote myocardium was, as much as possible, defined in a different coronary territory. In cases of disagreement, a third cardiovascular radiologist (F.P.), with 16 years of experience in cardiac CT, provided the final adjudication. All attenuation measurements were derived exclusively from iodine maps to ensure consistency across patients.

### Interpretation

MPI, CCTA, and LIE series were analysed and interpreted on a dedicated clinical workstation (Syngo.via VB60S, Siemens Healthineers) by two cardiovascular radiologists (X.G. and B.L.) with 11 and 12 years of experience in cardiovascular imaging. Coronary CT angiography findings were reported according to the Coronary Artery Disease Reporting and Data System (CAD-RADS 2.0).

**Figure 2:**
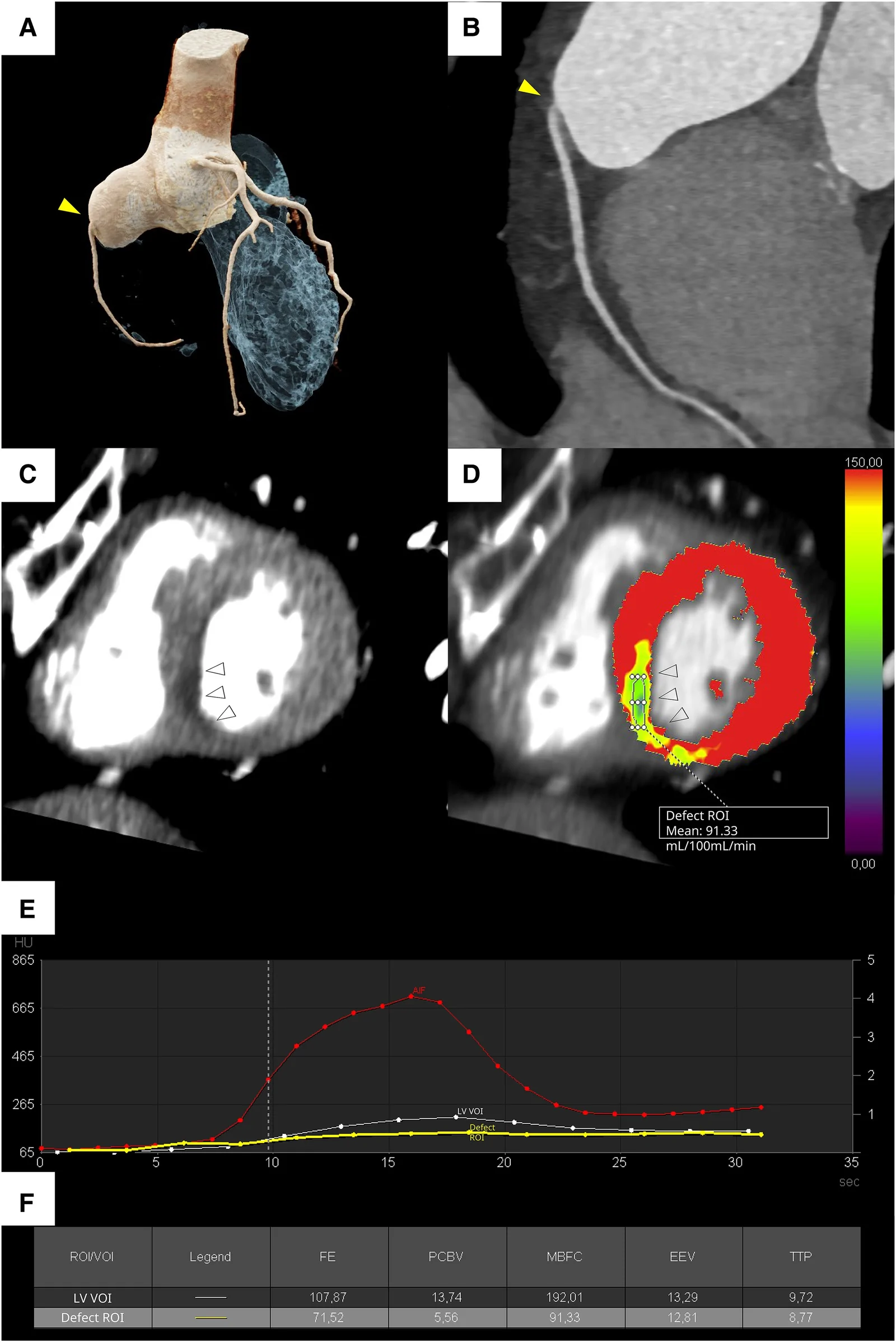
A 36-year-old woman with repaired ASO-TGA and neo-aortic root dilatation. (*A*) 3D VR CCTA: The yellow arrowhead indicates the origin of the reimplanted coronary artery with an acute take-off angle. **(***B***)** Curved multiplanar reformatted CCTA image demonstrating the proximal course of the coronary artery arising from the neo-aorta (arrowhead). **(***C***)** Short-axis CT myocardial perfusion image showing a relative hypoattenuating region within the left ventricular myocardium (arrowheads), suggestive of a perfusion abnormality. **(***D***)** Corresponding colour-coded myocardial blood-flow map demonstrating reduced myocardial perfusion in the affected territory (arrowheads). The colour scale represents myocardial blood-flow values. **(***E***)** Time–attenuation curves derived from dynamic EID-CT myocardial perfusion imaging, illustrating differences in contrast enhancement between arterial input function (red curve), normal myocardium (white curve), and the perfusion defect region (yellow curve). **(***F***)** Quantitative myocardial perfusion parameters derived from CT myocardial perfusion analysis, including MBF and related perfusion metrics comparing normal myocardium and the perfusion defect region. Coronary angiography confirmed CT findings, with a normal left coronary system and absent visualization of the right coronary artery ostium, likely related to backflow from the giant right coronary sinus aneurysm (S1).

**Figure 3:**
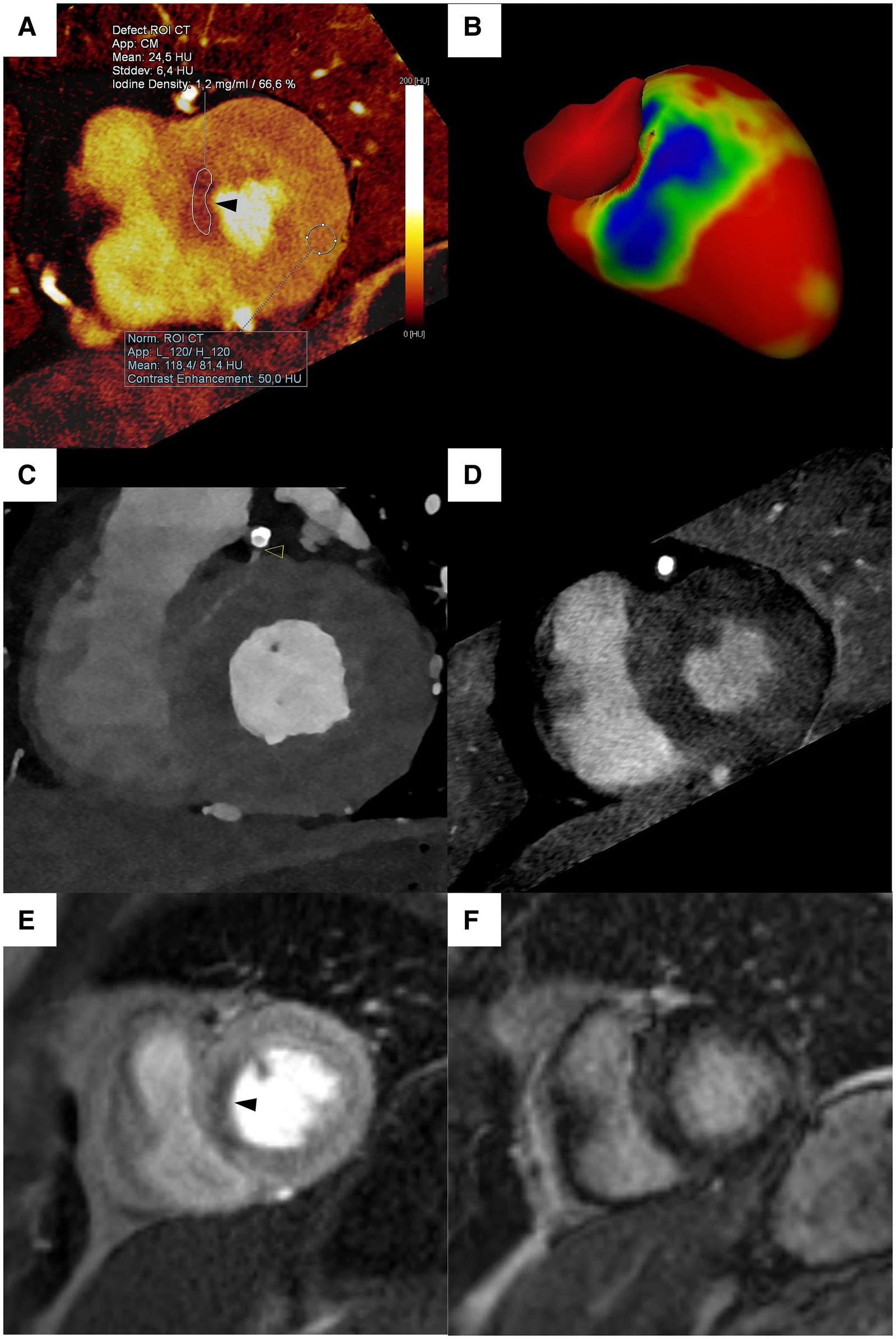
A 44-year-old man with repaired ASO-TGA. (*A*) Static stress PCCT myocardial perfusion image demonstrating a focal perfusion defect within the left ventricular myocardium (arrow). Quantitative analysis shows reduced iodine density within the defect compared with remote myocardium. **(***B***)** Three-dimensional myocardial perfusion map illustrating regional perfusion heterogeneity with a focal area of reduced perfusion (blue region). **(***C***)** CCTA showing the proximal course of the reimplanted coronary artery with an acute take-off angle from the neo-aorta (arrowhead). **(***D***)** Corresponding short-axis CT-MPI image demonstrates a relative hypoattenuating region consistent with a perfusion abnormality. **(***E***)** Stress-CMR image showing a perfusion defect in the corresponding myocardial territory (arrowhead). **(***F***)** LGE CMR image showing no corresponding myocardial scar.

CT myocardial perfusion imaging series were displayed using optimized window width and level settings automatically defined by a normalization region of interest drawn in the remote myocardium (‘CT perfusion’ and ‘Dual Energy Normalize Contrast’ tool, Siemens Healthineers). Images were reviewed in short-axis and orthogonal long-axis views, reconstructed as 5-mm-thick slices to improve signal-to-noise ratio. Myocardial blood flow of EID-CT measurements or mean iodine concentration and attenuation of PCCT scans in remote myocardium were calculated from regions of interest placed in the basal, mid, and apical left ventricular segments. Corresponding measurements were obtained in green colour maps, contrary to red colour remote myocardium or hypoattenuating regions, when present. Those areas were classified as ‘defect’. Representative cases of patients are illustrated in *Figures 2* and *3*. Additional images are shown in Supplementary data online, *Figure S1*

Double-oblique aortic root measurements and coronary arteries ostial kinking/angles were reported according to recent AHA/ESC guidelines.^4,17^

### Reference validation tests

Stress-CMR studies were performed on a 1.5-T system (MAGNETOM Sola FIT, Siemens Healthineers) using a comprehensive cardiac MRI protocol.^18^ Stress perfusion imaging was acquired after intravenous administration of 400 µg of regadenoson with first-pass myocardial perfusion imaging during pharmacologic stress and at rest. Late gadolinium enhancement imaging was performed 10 min after administration of a macrocyclic gadolinium-based contrast agent (Gadovist®, Bayer Healthcare) at a dose of 0.1 mmol/kg. Perfusion and late enhancement images were obtained in short-axis views covering the entire left ventricle from base to apex (contiguous 8-mm slices) and in long-axis views using a two-dimensional PSIR sequence.

Invasive coronary angiography was used as anatomic reference test and performed using standard femoral or radial access with selective coronary catheterization and quantitative assessment of coronary stenosis.

### Statistical analysis

Statistical analyses were performed using Stata version 17 (StataCorp LLC, College Station, TX). Continuous variables were expressed as mean ± standard deviation (SD) or median with interquartile range (IQR) depending on distribution assessed using the Shapiro–Wilk test, whereas categorical variables were reported as counts and percentages.

Diagnostic performance of CT-MPI was evaluated by calculating sensitivity, specificity, and diagnostic accuracy with 95% confidence intervals (CI). Analyses were performed at the per-segment level for comparison with stress CMR and at the per-vessel level using ICA as the reference standard. Because myocardial segments within the same patient are not independent observations, patient-level clustering was considered in the analysis to account for intra-patient correlation.

Because dynamic EID-CT MPI and static PCCT-MPI generate different perfusion parameters, continuous myocardial blood flow and iodine-concentration measurements were not combined. The diagnostic analyses used the final binary classification of each examination as positive or negative for a myocardial perfusion defect, based on the corresponding technique-specific interpretation criteria. In addition to the overall analysis, patient-level agreement with the reference diagnosis of myocardial ischaemia was assessed separately for the EID-CT and PCCT subgroups. Formal equivalence or non-inferiority testing between the two techniques was not performed because of the limited subgroup sizes and the small number of ischaemia-positive cases.

Interobserver and intraobserver reproducibility were assessed using intraclass correlation coefficients (ICCs) based on a two-way random-effects model with absolute agreement, with results reported together with 95% CI.

Associations between anatomical parameters and the presence of ischaemia were evaluated using univariable logistic regression, with results reported as odds ratios (OR) and 95% CI. Receiver-operating characteristic (ROC) curve analysis was used to assess the discriminatory performance of coronary stenosis, coronary take-off angle, and aortic diameter, with calculation of the area under the curve (AUC).

To explore determinants of exercise capacity, multivariable linear regression was performed with peak VO_2_ (%) as the dependent variable and coronary take-off angle, aortic root diameter, and coronary stenosis as independent variables.

Radiation dose parameters between scanner technologies were compared using the Mann–Whitney U test, given the non-normal distribution of dose–length product (DLP) values.^19^ All tests were two-sided, and a *P value <0.05* was considered statistically significant.

## Results

Thirty-six patients (29 men; 7 women) with a median age of 33.5 years (range: 19–47 years) and a median body mass index of 23.9 kg/m^2^ (range: 17.4–33.2 kg/m^2^) were included. Of the 36 included patients, 18 underwent static PCCT-MPI, and 18 underwent dynamic EID-CT MPI. The median aortic root diameter was 42 mm (38–45; range: 32–69 mm). Patient characteristics are described in *Table 1*.

**Table 1:** Characteristics of 36 patients with repaired transposition of the great arteries (TGA) who underwent a CT myocardial perfusion (CT-MPI)

Variables	Values
Male	29 (81)
Body mass index (Kg/m^2^)	24.0 ± 3.9 [17.4 –33.2]
Age (year)	33.5 ± 8.1 [19–47]
DLP-LIE (mGy.cm)	97 (78–126) [59.0 –217]
Thoracic pain	7 (19)
Dyspnoea	4 (11)
Palpitations/hyperexcitability	4 (11)
Obstructive coronary artery disease^a^	8 (22)
Coronary angle (°)	76.5 (41–92) [18–98]
Aorta diameter (mm)	42.0 ± 7.5 [32–69]
Arterial hypertension	1 (0.3)
Pulmonary hypertension	1 (0.3)
Atrial fibrillation	1 (0.3)
Diabetes	1 (0.3)
Arrhythmias	1 (0.3)
Abnormal ECG	8 (22)
D-dimers >1000 ng/mL	0 (0)
NT-proBNP (pg/mL)	186 ± 120 [25–620]
Peak high-sensitivity troponin (ng/L)	4.8 ± 3.1 [1–12]
VO_2_ (%)	87.5 ± 10.3 [66–106]
Medications	
Antiplatelets	7 (19)
B-blockers	9 (25)
Statins	1 (0.3)
ACE inhibitors	2 (5.6)
Endothelin receptor antagonist	1 (0.3)
Metformin	1 (0.3)

Qualitative variables are expressed as raw numbers followed by percentages into parentheses. Normally distributed quantitative variables are expressed as means ± standard deviations followed by ranges into brackets, and non-normally distributed quantitative variables are expressed as medians followed by first (Q1) and third (Q3) quartiles into parentheses and ranges into brackets.

^a^According to CAD-RADS 2.0 classification.

CT myocardial perfusion imaging demonstrated excellent diagnostic performance for ischaemia detection at the per-segment level. Of the 576 myocardial segments analysed, 22 (3.8%) were classified as ischaemic on stress CMR. CT myocardial perfusion imaging correctly identified 21 of these ischaemic segments, with one false-negative result, and correctly classified 552 of the 554 nonischaemic segments, with two false-positive results. Accordingly, CT-MPI achieved a sensitivity of 95.5% (95% CI, 77.2–99.9), a specificity of 99.6% (95% CI, 98.7–100.0), and an overall diagnostic accuracy of 99.5% (95% CI, 98.5–99.9) (*Table 2*).

**Table 2:** Diagnostic performance of CT-MPI compared with stress cardiac MRI (CMR) at the segment level and invasive coronary angiography at the vessel level

Analysis	Reference test	N (units)	Sensitivity % (95% CI)	Specificity % (95% CI)	Accuracy % (95% CI)
Per-segment Overall cohort	Stress CMR	576	95.5 (77.2–99.9)	99.6 (98.7–100.0)	99.5 (98.5–99.9)
Per-vessel Overall cohort	Coronary angiography	216	70.0 (34.8–93.3)	100.0 (98.2–100.0)	98.6 (96.0–99.7)
Per-coronary territory PCCT-MPI	Stress CMR	54	100.0 (39.8–100.0)	98.0 (89.4–99.9)	98.1 (90.1–100)
Per-coronary territory EID-CT MPI	Stress CMR	54	100.0 (15.8–100.0)	100.0 (93.2–100.0)	100.0 (93.4–100.0)

Significant coronary artery disease was defined as ≥50% luminal stenosis. Confidence intervals (CIs) were calculated using the exact binomial (Clopper–Pearson) method. McNemar exact test was used for paired comparisons.

For the stress-CMR territory-level analysis, the 16 myocardial segments were grouped into LAD, RCA, and LCx territories. Territory was considered positive when at least one constituent segment demonstrated a perfusion defect. Static PCCT-MPI and dynamic EID-CT MPI were interpreted using technique-specific criteria and analysed separately. The subgroup analysis was descriptive and was not designed to establish equivalence or non-inferiority between the two CT-MPI techniques.

At the coronary–territory level, 108 territories were evaluated, including 54 territories imaged with static PCCT-MPI and 54 with dynamic EID-CT MPI. Static PCCT-MPI correctly identified all four stress-CMR-positive territories and 49 of 50 stress-CMR-negative territories, corresponding to a sensitivity of 100% (95% CI, 39.8–100.0), specificity of 98.0% (95% CI, 89.4–99.9), and accuracy of 98.1% (95% CI, 90.1–100.0). Dynamic EID-CT MPI correctly classified all two stress-CMR-positive and all 52 stress-CMR-negative territories, corresponding to a sensitivity, specificity, and accuracy of 100% (95% CIs, 15.8–100.0, 93.2–100.0, and 93.4–100.0, respectively). The only discordant territory was a false-positive LCx territory on static PCCT-MPI; neither technique demonstrated a false-negative territory.

In per-vessel analysis using ICA, CT-MPI showed high sensitivity for the detection of obstructive coronary artery disease (70.0%, 95% CI: 34.8–93.3), together with excellent specificity (100.0%, 95% CI: 98.2–100.0) and high accuracy (98.6%, 95% CI: 96.0–99.7) as shown in *Table 2*.

In the modality-stratified patient-level analysis, CT-MPI classification agreed with the reference diagnosis of myocardial ischaemia in all patients in both subgroups. In the PCCT subgroup, all four reference-positive and all 14 reference-negative examinations were correctly classified. In the EID-CT subgroup, the two reference-positive examinations and all 16 reference-negative examinations were correctly classified.

Among symptomatic patients with normal stress echocardiography, CT-MPI was negative in 8 of 10 patients (80%), with no missed ischaemia on stress CMR. In symptomatic patients with abnormal or inconclusive stress echocardiography, CT-MPI was negative in three of six patients (50%) without false-negative findings compared with stress CMR or ICA. Among asymptomatic patients with normal stress echocardiography, CT-MPI was negative in 14 of 15 patients (93.3%). One patient in this group had ≥50% coronary stenosis on angiography despite negative CT-MPI and stress CMR. Overall, CT-MPI was negative in 30 of 36 patients (83.3%), with no significant difference between static and dynamic perfusion acquisitions (Fisher’s exact test; *P* = *0.34*).

### Interobserver and intraobserver agreement

Reproducibility was good to excellent for MBF measurements on dynamic EID-CT MPI and for mean myocardial attenuation and iodine–concentration measurements on static PCCT-MPI with interobserver ICCs of 0.83–0.88 and intraobserver ICCs of 0.88–0.91 (*Table 3*). Coronary measurements showed near-perfect agreement, with ICCs of 0.98 for coronary take-off angle and 0.99 for coronary stenosis.

**Table 3:** Intraclass correlation coefficients (ICCs)

Measurement	Interobserver ICC (95% CI)	Intraobserver ICC (95% CI)
Mean attenuation from PCCT-MPI	0.85 (0.74–0.91)	0.88 (0.78–0.93)
Mean iodine concentration from PCCT-MPI	0.83 (0.66–0.92)	0.90 (0.77–0.96)
MBF from EID-CTMPI	0.88 (0.76–0.94)	0.91 (0.80–0.96)
Coronary angle	0.98 (0.97–1.00)	0.97 (0.94–0.99)
Coronary artery stenosis	0.99 (0.98–1.00)	0.98 (0.95–0.99)

Intraclass correlation coefficients were calculated for the reproducibility of PCCT-MPI and EID-CT MPI and the coronary measurements, using a **two-way random-effects model with absolute agreement**, with results reported together with 95% confidence intervals.

For static PCCT-MPI, interobserver agreement for the presence or absence of myocardial ischaemia was almost perfect (Cohen’s κ, 0.85; 95% CI, 0.57–1.00), with an overall agreement of 94.4% (17 of 18 examinations).

### Anatomic and functional correlations

**Figure 4:**
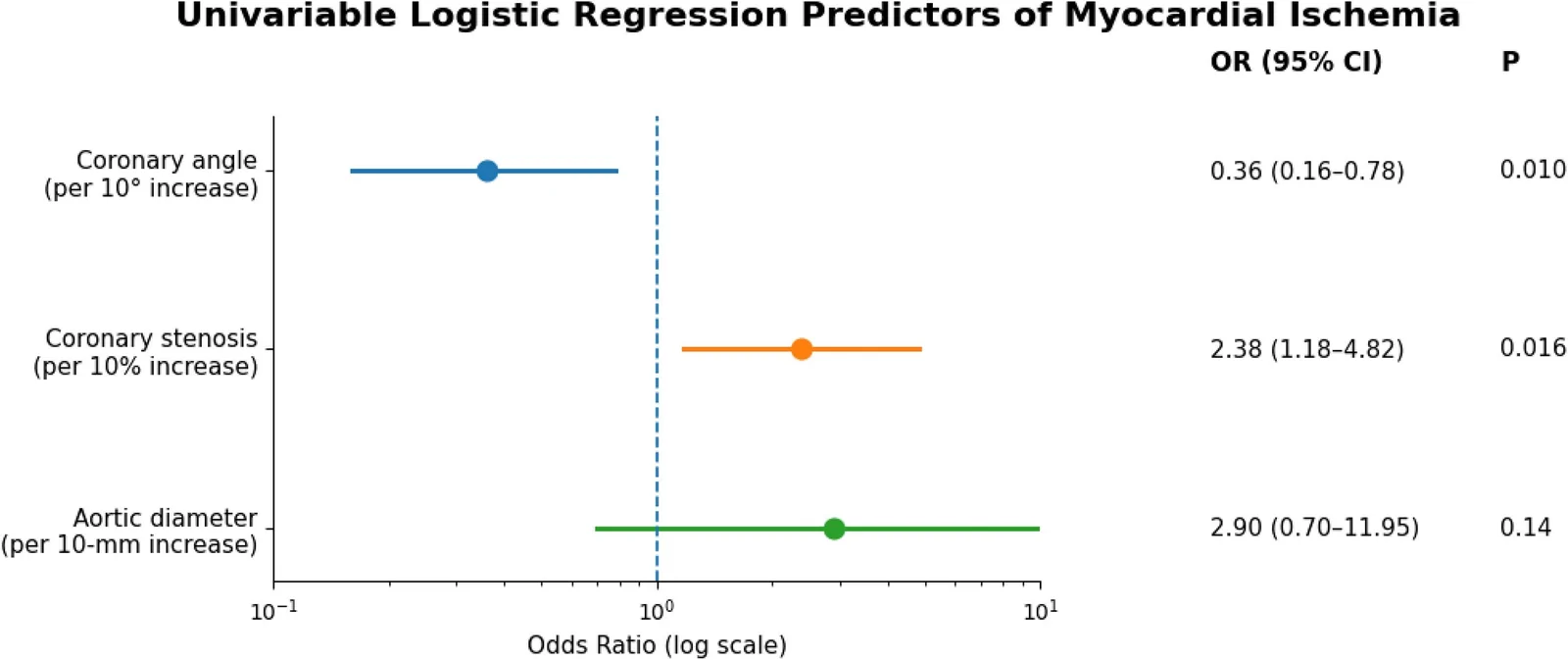
Forest plot of univariable logistic regression showing that coronary stenosis was associated with higher ischaemia risk, whereas a larger coronary take-off angle was protective. Aortic diameter was not significantly associated. Points represent odds ratios and bars indicate 95% confidence intervals.

**Figure 5:**
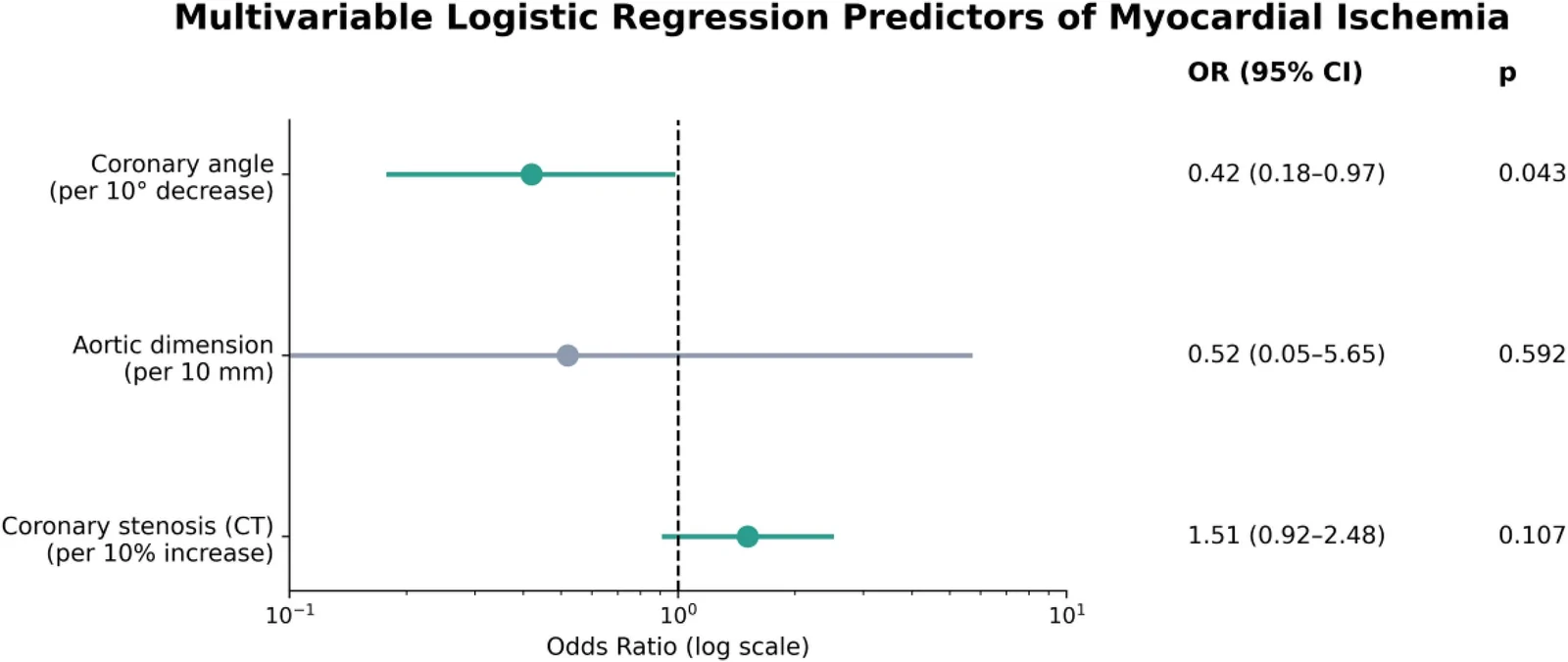
Forest plot of multivariable logistic regression showing that coronary take-off angle remained independently associated with myocardial ischaemia, whereas aortic dimension and coronary stenosis were not significant predictors. Points represent odds ratios and bars indicate 95% confidence intervals.

**Figure 6:**
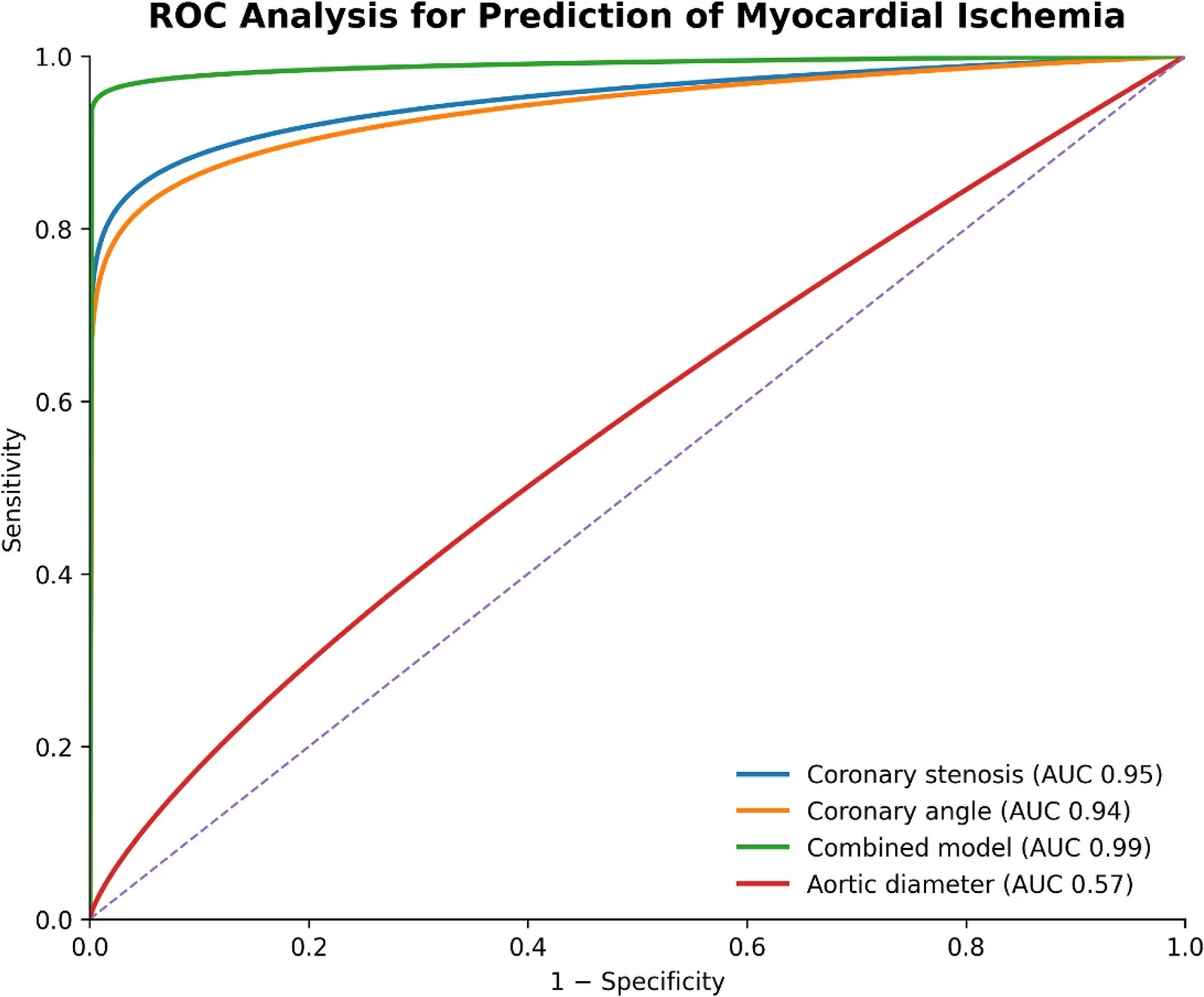
Receiver-operating characteristic curves showing the performance of coronary stenosis, coronary take-off angle, aortic diameter, and their combined model for predicting myocardial ischaemia. AUC values with 95% confidence intervals are shown.

Using per-patient median values from the three observers, univariable logistic regression identified coronary stenosis (OR 2.38, 95% CI 1.18–4.82*; P* = *0.016*) and coronary take-off angle (OR 0.36, 95% CI 0.16–0.78; *P* = 0.010) as significant predictors of myocardial ischaemia, whereas aortic diameter was not (*P* = *0.14*) (*Figure 4*). In multivariable analysis, coronary take-off angle remained the only independent predictor (*P* = *0.043*) (*Figure 5*). Receiver-operating characteristic analysis confirmed these findings. Coronary stenosis (AUC 0.95, 95% CI 0.87–1.00) and coronary angle (AUC 0.94, 95% CI 0.82–1.00) showed strong discrimination, whereas aortic diameter performed poorly (AUC 0.57, 95% CI 0.23–0.91). The combined model of coronary stenosis and angle yielded the highest diagnostic performance (AUC 0.99, 95% CI 0.94–1.00), and inclusion of aortic diameter did not improve discrimination (*P*  *<*  *0.001*) (*Figure 6*). A larger coronary take-off angle was also independently associated with higher exercise capacity (VO_2_%; β=0.28), whereas aortic root diameter (β=−0.21) and coronary stenosis (β=−0.03) were not significantly associated.

### Radiation dose metrics

The mean DLP of CT-MPI was 215 ± 123 mGy·cm (range: 50–569), corresponding to a calculated effective dose of 2.91 ± 1.7 mSv (range: 1.2–4.3). CT myocardial perfusion imaging radiation exposure was significantly lower with PCCT compared with EID-CT [150 mGy·cm (IQR, 51–280) vs. 281 mGy·cm (IQR, 170–419); *P* = 0.046].

**Figure 7:**
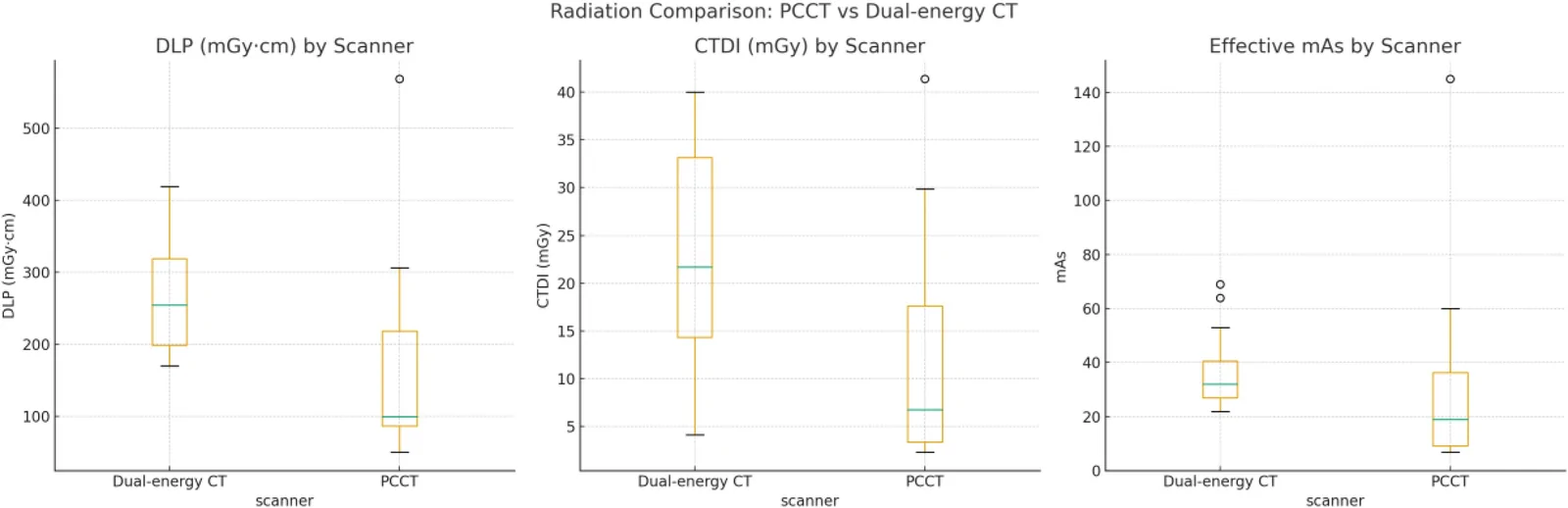
Radiation comparison between PCCT and EID-CT. Box plots showing DLP, CTDI, and effective tube current (mAs) for PCCT-MPI and EID. CT-MPI. Boxes represent interquartile ranges with medians, and whiskers indicate the full data range.

The mean CCTA DLP was 110.6 mGy·cm (range: 42–362 mGy·cm) for PCCT and 94.3 mGy·cm (range: 45–369 mGy·cm) for EID-CT, corresponding to estimated mean effective doses of 1.55 mSv (range: 0.59–5.07 mSv) and 1.32 mSv (range: 0.63–5.17 mSv), respectively (*P* = 0.91). Overall, radiation exposure was significantly lower with PCCT than with EID-CT, with a 39% reduction in mean DLP (*Figure 7*).

## Discussion

In this study, we evaluated the diagnostic performance of stress CT-MPI combined with CCTA in adults after ASO for transposition of the great arteries. To our knowledge, this represents the first validation of CT-MPI in a congenital heart disease population and specifically in adults after ASO-TGA.

Several important observations emerge. First, CT-MPI showed excellent diagnostic performance in detecting myocardial ischaemia in this population compared with stress CMR and ICA. Second, the integration of anatomical and functional information within a single CT examination could substantially reduce the need for additional ischaemia testing. Third, coronary take-off angle was strongly associated with ischaemia. Finally, photon-counting detector CT enabled a meaningful reduction in radiation exposure compared with conventional CT technology.

According to contemporary congenital heart disease guidelines, asymptomatic patients after ASO are recommended to undergo periodic CCTA as part of long-term surveillance to detect potential late coronary complications and high-risk anatomical features.^3,4,20^ In our results, CT-MPI allowed exclusion of myocardial ischaemia in asymptomatic patients, without missed ischaemia on stress CMR or significant coronary stenosis on ICA. These findings support the potential role of CT-MPI as a comprehensive baseline examination in young adults after ASO, particularly in patients seeking clearance to progressively increase their physical activity under the supervision of congenital cardiology specialists.^20–23^

As most patients who underwent ASO for d-TGA are now reaching young adulthood, CT-MPI may represent a valuable noninvasive strategy for ischaemia evaluation, particularly in patients younger than 40 years, a population in which the prevalence of atherosclerotic coronary artery disease remains very low.^24^ In this setting, CT-MPI may further reduce the need for invasive testing when ischaemia is suspected. As this population ages and conventional cardiovascular risk factors emerge, larger prospective studies should assess the role of CT-MPI for the comprehensive evaluation of both congenital and acquired coronary disease, compared to established functional tests.

In line with previous studies, coronary geometry and stenosis were strongly associated with the presence of ischaemia.^5–7,25^ This suggests that CT-MPI could be selectively integrated into clinical practice when high-risk anatomical features—such as an acute coronary take-off angle—are identified or suspected with CCTA. In such cases, the addition of stress perfusion imaging may help determine the functional significance of anatomical findings and assist experienced cardiac imagers in refining clinical decision-making within routine follow-up.

In our study, neo-aortic root dilatation was not strongly associated with coronary stenosis or ischaemia in our cohort, consistent with previous literature.^5–7^ Echocardiography remains the first-line imaging modality for surveillance of neo-aortic root dimensions and aortic valve function after ASO-TGA. However, in cases of progressive neo-aortic growth and important aortic regurgitation and/or the presence of clinical symptoms, CT-based imaging may provide complementary anatomical and functional information. In such scenarios, CT-MPI could contribute to a more comprehensive evaluation prior to potential surgical or interventional management.

Despite the increasing availability of congenital heart disease surgery and advances in imaging technologies, access to specialized expertise and multidisciplinary care remains heterogeneous across centres.^3,26^ Consequently, diagnostic pathways often involve multiple sequential tests. By potentially reducing the need for downstream investigations, emerging CT technologies may offer a comprehensive ‘one-stop’ examination with acceptable radiation exposure. With the growing availability of photon-counting detector CT across different vendors, its improved spatial resolution and enhanced spectral capabilities may further expand the role of CT in this setting.^27,28^ As shown by increasing evidence in the literature, PCCT technology may provide a valuable alternative in patients who cannot undergo cardiac MRI, have pacemakers, or have limited access to it, allowing simultaneous evaluation of myocardial ischaemia and myocardial scar with reasonable radiation doses.^27–29^

When each CT-MPI technique was interpreted using its corresponding predefined criteria, the modality-stratified patient-level analysis demonstrated agreement with the reference diagnosis of myocardial ischaemia in both subgroups. As expected, static perfusion imaging acquired with photon-counting CT was associated with significantly lower radiation exposure—approximately 40% lower—than dynamic CT-MPI. Although not the primary objective, this study also provides one of the first validations of stress CT-MPI using photon-counting CT detectors.^30^ Because only six patients had myocardial ischaemia, these findings represent descriptive agreement and should not be interpreted as demonstrating formal equivalence between the two CT-MPI techniques.

Despite its novelty, we acknowledge that this study has several limitations. First, this is a retrospective single-centre study with a moderate sample size, partly explained by the exclusion of minor subjects, which may limit the statistical power of the analysis. Moreover, two CT–stress perfusion techniques were used, which may introduce protocol heterogeneity. Although CT-MPI showed strong potential for ruling out ischaemia, the exceptionally high segment-level accuracy should be cautiously interpreted in the context of the low prevalence of ischaemic segments. Finally, future studies incorporating invasive physiological measurements (FFR/iFR) are warranted to provide a more robust functional reference standard. As traditional noninvasive imaging techniques are increasingly enriched with advanced quantitative methods, FFR-CT could also be incorporated into further evaluations and comparisons of CT-MPI in future studies.

## Conclusion

Stress CT-MPI combined with CCTA was feasible and demonstrated high diagnostic performance for detecting myocardial ischaemia in adults after ASO for transposition of the great arteries. These preliminary findings suggest that stress CT myocardial perfusion may provide a valuable one-stop examination, particularly in symptomatic patients with known coronary abnormalities when stress CMR is unavailable, contraindicated, or inconclusive. Larger prospective studies are required to validate these findings and to define its role in routine clinical workflows and its potential impact on downstream testing.

## Supplementary Material

qyag139_Supplementary_Data

## Data Availability

The data underlying this article will be shared on reasonable request to the corresponding author.

## Lead author biography

Dr. Christos Gkizas is a Radiology Consultant and Clinical MRI Director at the Cardiovascular and Thoracic Imaging Unit of the Heart and Lung Institute, University Hospitals of Lille, under the leadership of Prof. François Pontana. His research primarily focuses on biomarkers in cardiac MRI and photon-counting CT, applied to noninvasive cardiovascular imaging. Dr. Gkizas is an active member of several international scientific committees, serving on the Translation Committee of the Society for Cardiovascular Magnetic Resonance (SCMR) and the Educational Committee of the European Society of Cardiovascular Radiology (ESCR).
