# Supplementary material for: Stress CT myocardial perfusion imaging after arterial switch operation for transposition of the great arteries
Source: Eur Heart J Imaging Methods Pract. 2026 Aug 25;4(3):qyag139. doi: 10.1093/ehjimp/qyag139 (PMC13521289; doi:10.1093/ehjimp/qyag139)
Supplement: Supplementary file 1 [file qyag139_supplementary_data.docx]

Coronary angiography: No visualization of the RCA ostium, likely related to backflow from the giant right coronary sinus aneurysm.
